# Public Knowledge, Attitudes, and Practices Toward COVID-19 Pandemic: A Cross-Sectional Study in Delhi and National Capital Region (NCR), India

**DOI:** 10.1177/21582440231153370

**Published:** 2023-02-24

**Authors:** Upma Gautam, Deeksha Bajpai Tewari

**Affiliations:** 1 University School of Law and Legal Studies, Guru Gobind Singh Indraprastha University, Delhi, India; 2 University of Delhi, India

**Keywords:** COVID-19, knowledge, attitude, practices, lockdown, social distance

## Abstract

To assess knowledge, attitudes, and practices of people toward coronavirus disease 2019 (COVID-19) pandemic in National Capital Territory of Delhi and National Capital Region (NCR), India. Various nations, including India, devised strategies to impose lockdowns and movement restrictions on their citizens in order to mitigate the effects of COVID-19. Cooperation and compliance by the populace are crucial to the effectiveness of such measures. People’s knowledge, attitudes, and behavior toward such diseases are crucial in determining the adaptability of a society to such changes. Using Google Forms, a self-designed semi-structured questionnaire was created. This study is cross-sectional. Participants were eligible to participate if they were over the age of 18 and presently resided in the study area. The questionnaire included demographic variables such as gender, age, location, occupation, and income level. A total of 1,002 people completed the survey. A 48.80% of the respondents in the study group were females. The mean knowledge score was 13.14 (Maximum Score = 17), while the mean attitude score was 27.24 (Maximum Score = 30). Most of the respondents (96%) had adequate knowledge about the disease’s symptoms. A 91% of the respondents had an average attitude score. A 74.85% of respondents accepted that they had avoided large social events. Gender had a negligible impact on the average knowledge score, while the score differed significantly across education levels and occupation categories. Consistent dissemination of messages regarding the virus, its spread, control measures implemented, and precautions expected from the public aids in reassuring the public and reducing their anxiety regarding the virus.

## Introduction

In the final month of 2019, a pathogenic human coronavirus, SARS-CoV-2, coronavirus disease 2019 (COVID-19), was identified in Wuhan, the provincial capital of China’s central Hubei province (Holshue et al., 2020). In addition, on January 11, China reported the first COVID-19-related death in a 61-year-old male exposed to the seafood market (World Health Organization [WHO], 2020d). In a matter of weeks, the infection spread rapidly across the globe. This virus has caused numerous illnesses and deaths since then (WHO, 2020a). The International Health Regulations (2005) Emergency Committee of the World Health Organization (WHO) declared the novel coronavirus disease (COVID-19) an international emergency of public health concern on January 30, 2020 (Wadhwani et al., 2020). The World Health Organization changed this status to pandemic on March 11, 2020, when the virus had spread to 114 countries (WHO, 2020e). Since then, the mortality rate associated with the current pandemic has increased. On December 12, 2020, nearly a year after the detection of the first case of COVID-19 in the world, there were 69,143,017 confirmed cases and 1,576,007 deaths worldwide (WHO, 2020c). By the middle of September 2021, there were approximately 21.9 billion (219 million) confirmed cases worldwide, and the death toll had risen to 4.55 million (Dong et al., 2020).

India reported the first confirmed case of COVID-19 on January 30, 2020. An individual from Wuhan, China, made the journey. On February 14, 2020, he recovered fully from his infection (Balasubramanian & Balakrishnan, 2020).

Since then, India’s government has been on high alert and is continually monitoring the spread of the disease. It is further developing effective strategies for curtailing the infection in line with the WHO guidelines (Ministry of Home Affairs [MHA], 2020). After a 14-hour voluntary exercise of “*Janta Curfew*,” or public curfew, India imposed a nationwide lockdown from March 25 to May 31, with a few exceptions in different states (MHA, 2020). This was India’s first announced 21-day lockdown (March 25–April 14, 2020). This only allowed essential services to operate for the entire 130 million inhabitants (Singhal, 2020). Simultaneously, the Central and State governments began the arduous tasks of establishing isolation and quarantine centers; lab services; research and development; the deployment of medical staff; and the streamlining of PPE procurement (Kong et al., 2020; Wilder-Smith & Freedman, 2020). These measures were crucial for flattening the epidemic curve and preventing a possible future outbreak (Pan et al., 2020; WHO, 2020b). In India, the fight against COVID-19 is still ongoing. As of mid-September 2021, this pandemic has caused 3.35 crore (30.35 million) confirmed cases and approximately 4.45 lakh (0.45 million) fatalities in India (Hazem et al., 2020).

Delhi and the National Capital Region (NCR) encompass approximately 21,200 m^2^ and have a population density of nearly 2,200 inhabitants per square meter. As of mid-September 2021, Delhi has a staggering 14.38 million confirmed cases and 25,085 fatalities. In April and May of 2021, the second wave of the pandemic that affected this region caused the most fatalities and confirmed cases to be reported. Individual and community-level adherence to infection protection and control (IPC) measures has a significant impact on the dynamics of contagion transmission. The study area is characterized by zones that have the potential to act as hotspots for the spread of disease due to steep obstacles to social isolation and a lack of basic sanitation amenities such as access to clean water and soap for handwashing caused by a dense population. Informal settlements and worker areas pose a universal obstacle to the strict implementation of COVID-19 protocols. In these regions, access to essential services such as food, water, sanitation, and medicine is inconsistent and unequal. The study area is also the country’s largest metropolitan area. It attracts a large migrant population from various states, which further complicates the issues of following contact tracing protocols, isolating infected individuals, and imposing quarantine (Pan et al., 2020).

Governments around the world have taken unprecedented national measures to combat the spread of the virus, but the success or failure of these measures is intrinsically tied to public behavior. Importantly, people’s adherence to government-established preventive measures is essential to halting the spread of the disease. The relationship between public knowledge and attitudes toward COVID-19 and their adherence to recommended preventive measures is positively correlated. According to studies, public awareness is crucial for combating pandemics (Al-Hanawi et al., 2020; Chirwa, 2020). It is also stated that a person’s understanding of the disease and likelihood of adopting positive practices must be enhanced by factors such as the disease’s severity, susceptibility to it, and structural barriers that may prevent him or her from adopting positive practices. This pertinent data was collected through various types of cross-sectional surveys, with knowledge, attitude, and practice (KAP) surveys being the most common. Knowledge-Attitudes-Practices (KAP) quantitative research is a standard method for understanding and analyzing human responses to particular phenomena, particularly in health studies (Launiala, 1970; Manderson & Aaby, 1992; Manderson & Levine, 2020).

A KAP survey means **K**nowledge, **A**ttitude, and **P**ractices study. A KAP survey is a quantitative type method (predefined questions and formatted in standardized questionnaires) that provides access to quantitative and qualitative information (H e Ka P, 2011). The respondents’ knowledge and attitudes are the focus of these studies. These questions are designed to elicit key knowledge, social skills, and know-how about issues that are commonly shared by a population or target group. These studies can play a significant role in identifying the need to enhance the knowledge, attitude, and practices around specific health issues through building an understanding of what is known and done about various subjects relating to health. They also establish a baseline (reference value) for future assessments and can help measure the effectiveness of health education activities in changing health behaviors.

Such KAP studies provide insight into the essentially required interventions to address the public’s possible misconceptions about the pandemic. They can further play a crucial role in the development of preventive strategies and formulating health promotion programs. Lessons learned from the studies conducted during the past pandemics pointed out the critical negative role that stigma, panic, and emotion associated with the disease can play in outlining an effective combat strategy to contain the disease’s spread (Li et al., 2020). These studies can also act as a blueprint for the Governments in addressing future health crises involving infectious diseases.

During pandemics, educating, engaging, and mobilizing the public to become active participants may aid in achieving public health emergency preparedness, thereby reducing the vulnerability of the entire population(Lee & You, 2020). Recent studies have demonstrated that COVID-19 morbidity and mortality rates can be significantly reduced when individuals engage in preventive behaviors, such as practicing personal hygiene and maintaining social distance (Anderson et al., 2020; Duhon et al., 2020). Therefore, the public must adopt the practice of routinely exercising caution, and this has become a new norm as well. For the promotion and maintenance of preventive behaviors in the general population, an indication of the social, cognitive, and psychological factors associated with the behaviors is necessary. Prior research on infectious disease epidemics revealed that awareness and knowledge (Aburto et al., 2010; Brug et al., 2004; Lin et al., 2014), perceptions of risk (Aburto et al., 2010; Anderson et al., 2020), and effectiveness beliefs (Lee & You, 2020) motivate individuals to engage in preventive behaviors. Similarly, recent research on COVID-19 revealed that knowledge (Azlan et al., 2020; Rahman & Sathi, 2020; Saefi et al., 2020), realized controllability (Azlan et al., 2020; Zhong et al., 2020), optimistic beliefs (Saefi et al., 2020), emotions (Saefi et al., 2020), and risk perception (Honarvar et al., 2020) may all contribute to the general public’s precautionary behavior. Several KAP studies have examined the relationships between knowledge and attitudes or behaviors beyond the prevalence of each. A higher level of knowledge is positively associated with the practice of preventive measures and attitudes are also positively associated with preventive behaviors. Nonetheless, the majority of these studies examined the direct effects of knowledge on practicing preventive behaviors (Afzal et al., 2021; Alrubaiee et al., 2020; Lau et al., 2020; Tamang et al., 2020) or attitudes without investigating the indirect effects of knowledge on practices mediated by attitudes to explain the psychological mechanism underlying how individuals engage in behaviors based on their health knowledge. Little is known about the indirect influence of knowledge on practices via attitudes in the context of COVID-19. The connection between people’s attitudes and practices is well established in psychology, explained through the Theory of Planned Behavior (Ajzen, 2011). Internationally derived links between health-related knowledge, attitudes, and practices are used to inform strategies and policies intended to improve health (Tolvanen et al., 2009). During the pandemic of COVID-19 in India, behavioral factors and associated vulnerabilities are poorly documented. As described in the preceding section, such studies are available for a variety of nations and population groups from various regions of the world. This study investigates whether the general population engages in precautionary behaviors recommended by national guidelines and behavioral interventions, as well as which populations should receive priority in health behavior change interventions. We quantified and investigated the relationships between knowledge, attitudes, and practices, as well as the interactions between sociodemographic variables and knowledge, attitude, and practice components.

## Objectives of the Study

i. To assess knowledge, attitudes, and practices of people toward coronavirus disease 2019 (COVID-19) pandemic in Delhi and NCR, India during 2020.
ii. To understand the initial response of the people of Delhi, NCR toward COVID-19 and to assess the success of the government’s health campaigns during the year 2020.

## Materials and Methods

### Participants and Data Collection

We used an anonymous online survey with a cross-sectional design to evaluate the public’s knowledge, attitudes, and behaviors during the COVID-19 epidemic. This cross-sectional study was conducted from 1 to 10 December 2020 among residents of Delhi and the National Capital Region (NCR). In accordance with social distancing protocol, the survey was conducted online via the Google form platform. The link to the Google form was distributed via multiple WhatsApp groups and emails. Residents over the age of 18 who currently reside in Delhi and the National Capital Region (NCR) were eligible to participate in the survey. Different strategies were employed to reach as many respondents as possible in the study area. This included utilizing the personal and professional networks, community leaders, and workplace colleagues of the researchers. Using a combination of purposive and snowball techniques, the respondents were chosen. Since an online survey was conducted in English, the results are restricted to the demographic group of adults over 18 who could comprehend the questionnaire in English. The availability of Internet connectivity and mobile connectivity was also a factor. Because the survey was conducted online and there were no face-to-face meetings with respondents, only those with access to the language and internet connectivity were considered limiting factors.

On the first page of the online questionnaire, the researchers informed the participants about the study’s history and objectives. Additionally, they were informed that their identities would be kept secret. Participants gave informed consent to participate in the survey. Participants’ informed consent was a preliminary section of the questionnaire. Participants had to respond to a yes/no question to confirm their willingness to participate voluntarily. After confirming the question, the participant was instructed to fill out the self-reported questionnaire. The participants also had the option to withhold their identities.

### Questionnaire

Based on the findings of previous COVID-19 studies’ literature reviews (Dkhar et al., 2020; Iorfa et al., 2020; Zhong et al., 2020) and the explanation of COVID-19 found on the WHO’s website, the questionnaire questions were developed (WHO, 2020a). The questionnaire contained four distinct sections. The first section discussed the socio-demographic characteristics of the respondents, including age, gender, marital status, level of education, employment status, and income. The second section asked respondents about their familiarity with COVID-19. This section contained seventeen questions regarding the disease, its transmission, characteristics, symptoms, isolation, prevention, and risk groups. The responses to these questions were true or false, and I do not know as alternatives. A correct response received one (1) point, while a false or don’t know response received zero points. The maximum score was between 0 and 17. A higher score indicated greater COVID-19 knowledge. The third section assessed the attitudes of participants toward COVID-19. The researchers used a Likert scale with five points. On a scale ranging from “strongly disagree” to “strongly agree,” respondents were asked to indicate their level of agreement with each of these statements. This section included questions designed to gauge respondents’ confidence in the government’s capacity and strategy to combat the pandemic. The scores were calculated by averaging the responses to the six questions. The range of total scores was from 6 to 30, with high scores indicating favorable attitudes. In other global studies evaluating knowledge, attitudes, and practices regarding COVID-19 (Hussain et al., 2020; Khasawneh et al., 2020; Saqlain et al., 2020; Srichan et al., 2020), authors have categorized population attitude scores into low, medium, and high categories. The final section consisted of questions regarding practice and conduct during the pandemic. There were only two options for questions about COVID-19 practice: “yes” or “no.” Using the Rasch model of measurement, the questionnaire was revalidated. The questionnaire’s reliability and validity were determined to be satisfactory, with real item reliability (RMSE) values of 0.98 for the attitude scale, 0.98 for the knowledge scale, and 0.99 for the practice scale.

### Statistical Analysis

The Statistical Package for the Social Sciences (SPSS) version 26 was used to analyze the data in this study. Frequencies and percentages were used in descriptive statistics. To determine the differences between groups for specified demographic factors, the researchers utilized chi-square tests, independent samples *t*-tests, and one-way analysis of variance (ANOVA). The level of statistical significance was fixed to *p* = .05. The internal reliability of the Likert scales used to assess respondents’ attitudes was determined using Cronbach’s alpha. Cronbach’s alpha coefficient was .81, indicating a high degree of internal consistency. A Shapiro-Wilk’s test and a visual inspection of their histograms, *Q–Q* plots, and box plots showed that the knowledge scores were having skewness −0.76 (*SE* = 0.04; negatively skewed) and kurtosis 1.09 (*SE* = 0.09). On the other hand, Attitude scores were having skewness −1.83 (*SE* = 0.084; negatively skewed) and kurtosis 7.90 (*SE* = 0.167). A Cronbach’s alpha coefficient reliability test was used to determine the internal consistency of the data about the attitude measures. Cronbach’s alpha coefficient for attitude scores was .67. van Griethuijsen et al. (2015) stated that the range of Cronbach’s alpha between .6 and .7 is considered adequate and reliable. Thus, items regarding the measure of attitude on COVID-19 are acceptable. In this study, the Relative Importance Index method is used to analyze the survey results regarding the attitudes and responses of respondents toward COVID-19. It is a weighted average method in which the average rank for each question is calculated, and the rank for each attitude response is derived from the average rank of the questions grouped under that attitude response. Based on the questions in the third section of the questionnaire, the attitude of the respondents toward COVID-19 has been evaluated. To maintain respondent anonymity, the responses of the entire dataset were numerically coded. Both the data file and the statistical analysis were stored in separate files. The key to code linkage information was stored separately from the statistical analysis data file.

## Results

### Demographic Characteristics

There were 1,412 responses to the survey. There were a total of 1,002 responses included in the study. Due to insufficient survey responses or failure to meet age requirements, 410 responses were omitted. A 513 (51.19%) of the total respondents were male, while 489 (48.80%) were female. Almost 41% of respondents were between the ages of 18 and 29. Approximately 36% of respondents were unemployed. A 24.55% of respondents lived in the southwest region of Delhi. Included among the “others” marital statuses are divorced, separated, etc. Education Levels marked as “others” include levels of education beyond post-graduation as well as those who do not possess a bachelor’s degree but have completed professional courses. Other demographic characteristics are detailed in Table 1.

**Table 1.** Socio-Demographic Profile.

Variables	*N* = 1,002	Percentage (%)
Gender		
Male	513	51.19
Female	489	48.80
Age category (years)		
18–29	408	40.71
30–39	168	16.76
40–49	198	19.76
50–59	129	12.87
60 and above	99	9.88
Marital status		
Married	711	70.95
Not married	270	26.94
Others	21	2.09
Education level		
High school or below	321	32.03
Graduate	423	42.21
Postgraduate	180	17.96
Others	78	7.78
Work status		
Government employee	249	24.85
Private sector employee	204	20.35
Retiree	36	3.59
Self-employed	150	14.97
Unemployed	363	36.22
Monthly income		
Less than 20,000	360	35.92
20,000–50,000	210	20.95
50,000–1 lakh	219	21.85
More than 1 lakh	213	21.25
Locality		
North Delhi	39	3.89
Northwest Delhi	78	7.78
New Delhi	54	5.38
South Delhi	102	10.17
Southwest Delhi	246	24.55
Central	27	2.69
Shahdara	12	1.19
East Delhi	87	8.68
Northeast Delhi	12	1.19
Southeast Delhi	18	1.79
West Delhi	114	11.37
Gurgaon	105	10.47
Noida	81	8.08
Greater Noida	21	2.09
Faridabad	6	0.59

### Assessment of Knowledge

The study used seventeen questions to measure the respondents’ knowledge of COVID-19. The respondents’ average knowledge score was 13.14 (*SD* = 1.56, Maximum score = 17, and Minimum Score = 5). About 77.29% of the respondents had average knowledge about COVID-19. There was no significant difference between the male average knowledge score (13.145) and the female average knowledge score (13.148). The highest proportion (99.40%) of respondents were aware of the importance of avoiding crowded places to curb the virus’s spread. A significant proportion (97.60%) of respondents had good knowledge about the importance of isolation in reducing the spread of COVID-19. A large proportion of respondents were knowledgeable about the virus’s symptoms and were clear that early identification and action would prevent mortality. The participants’ responses were so coded that every correct answer was accorded a “1,” and every wrong answer was accorded a “0.” The correct answers are bold in Table 2.

**Table 2.** Participant Knowledge of COVID-19 (*N* = 1,002).

Questions	True (%)	False (%)	I don’t know (%)
01. Fever, fatigue, a dry cough, and body aches are the most common clinical symptoms of COVID 19.	960 (95.80)	15 (1.49)	27 (2.69)
02. Stuffy nose, runny nose, and sneezing are less common in people infected with the COVID 19 virus, as opposed to the common cold.	519 (51.79)	288 (28.74)	195 (19.46)
03. There is no effective cure for COVID-19 at the moment, but early symptomatic and supportive treatment can help most patients recover.	921 (91.91)	63 (6.28)	18 (1.79)
04. Not everyone who has COVID-19 will acquire severe symptoms. Only individuals who are elderly and have chronic illnesses are at an increased risk of developing severe instances.	792 (79.04)	162 (16.16)	48 (4.79)
05. Consumption of wild animals or contact with them would result in infection with the COVID-19 virus.	69 (6.88)	774 (77.2)	159 (15.86)
06. When a person with COVID-19 does not have a fever, they can transmit the virus to others.	849 (84.73)	69 (6.88)	84 (8.38)
07. COVID-19 virus spreads via infected persons’ respiratory droplets.	930 (92.81)	15 (1.49)	57 (5.68)
08. The COVID-19 virus is spread through the air.	519 (51.79)	291(29.04)	192 (19.16)
09. COVID-19 spreads from person to person within close proximity (approximately six feet)	873 (87.12)	75 (7.48)	54 (5.38)
10. COVID-19 can be contracted by touching a virus-infected surface or object and then touching one’s nose, mouth, or eyes.	972 (97.0)	3 (0.29)	27 (2.69)
11. Antibiotics are a successful COVID-19 therapy.	300 (29.94)	372 (37.12)	330 (32.93)
12. Ordinary residents can protect themselves from infection with the COVID-19 virus by wearing masks.	948 (94.61)	33 (3.29)	21 (2.09)
13. Children and young adults must take additional efforts to avoid infection with the COVID-19 virus.	858 (85.62)	111 (11.07)	33 (3.29)
14. Individuals should avoid congested areas such as train stations, markets, and so on to avoid contracting the COVID-19 virus.	996 (99.40)	3 (0.29)	3 (0.29)
15. Isolation and treatment of patients infected with the COVID-19 virus are efficient methods of containing the virus’s transmission.	978 (97.60)	18 (1.79)	6 (0.59)
16. Individuals who have come into touch with an infected person should be isolated immediately for a minimum of 14 days.	951 (94.91)	30 (2.99)	21 (2.09)
17. Consumption of nutritious foods and water strengthens the body’s immunity and resistance to COVID-19.	909 (90.71)	42 (4.19)	51 (5.08)

The following non-parametric tests were used to determine the significance of the variance in knowledge scores across demographic variables: Mann-Whitney Test, Kruskal-Wallis Test There was no significant difference in knowledge scores between men and women (*p* = .05). The knowledge tests vary considerably between age groups (*p* = .05). A 50-to-59-year-old respondents had the lowest average knowledge score, 12.96. The age group of 40- to 49-year-old respondents had the highest knowledge score (13.43). The COVID-19 knowledge scores varied significantly by marital status (*p* = .05). The average knowledge score of married respondents (13) was significantly lower than the average knowledge score of unmarried respondents (13.46). There was a significant difference between education level and knowledge scores. The average knowledge score ranged between 13.0 and 13.41 for respondents with graduate and postgraduate education levels, respectively. There was a significant difference between workers and non-workers in various sectors. In addition, local and regional residents’ knowledge scores differed significantly.

### Assessment of Attitudes

Six questions were posed to the respondents to determine their perspectives on COVID-19-specific protocols and behaviors. Positive attitudes toward strict adherence to these protocols would pave the way for preventing the spread of the virus. The mean attitude score of respondents was 27.24 (*SD* = 2.70, maximum score = 30, and minimum score = 9). Approximately 91% of respondents had an average attitude. Following the established protocols of social distancing, hand-washing, etc., an overwhelming majority of respondents displayed a highly positive attitude toward criticality. The attitudes of respondents regarding the effectiveness of strict COVID-19 measures enacted by governments to combat the spread of the virus were not overwhelmingly positive. Since the responses were scored using the Likert Scale, the relative importance of each statement was determined using the relative importance index method. The Relative Importance Index (RII) is a non-parametric method commonly employed by logistics and management researchers to analyze structured questionnaire responses for data involving ordinal measurement of attitudes. In terms of assigning relative importance to the statements, respondents gave the most weight to the necessity of hand washing to prevent the disease, followed by social isolation as an essential prevention strategy. The respondents exhibited scepticism regarding the country’s adoption of stringent measures to control COVID-19 as a national strategy. The respondents were the least optimistic about the eventual containment of the disease. This can be partially explained by the unavailability of the vaccine, which has shaken the respondents’ faith in eventual disease control. Although those who believed the measures would be successful in controlling the spread of the virus outnumbered those who disagreed that the country would eventually control the spread of the virus, their numbers were not as conclusive as those of other attitude questions (Table 3).

**Table 3.** Attitudinal Responses Toward COVID-19.

Statements	Strongly disagree	Disagree	Neutral	Agree	Strongly agree	Relative important index (RII)	Rank
	*N* (%)						
I must maintain a safe distance from people to avoid transmitting COVID-19	4 (0.39)	3 (0.29)	12 (1.19)	57 (5.68)	926 (92.41)	0.978842	2
Hand washing is critical to protecting me against COVID-19	3 (0.29)	3 (0.29)	4 (0.39)	46 (4.59)	946 (94.41)	0.98503	1
To avoid exposure to COVID-19, I should stay at home if I am ill	13 (1.29)	3 (0.29)	12 (1.19)	63 (6.28)	911 (90.91)	0.970459	3
COVID-19 will eventually be controlled successfully	25 (2.49)	25 (2.49)	286 (28.4)	266 (26.54)	400 (39.92)	0.797804	6
The country’s tough measures may contribute to the country’s victory over COVID-19	25 (2.49)	34 (3.39)	187 (18.66)	229 (22.85)	527 (52.59)	0.839321	5
Adherence to the precautions established by the Ministry of Health will help prevent the spread of COVID-19	7 (0.69)	22 (2.19)	133 (13.27)	289 (28.84)	551 (54.99)	0.870459	4

There was no significant difference between male attitude scores and female attitude scores (*p* ≤ .05). The average female attitude score was 27.37 (*SD* = 2.70, maximum score = 30, and minimum score = 9). Average male attitude score was 27.11 (*SD* = 2.70, maximum score = 30, and minimum score = 9). Attitude did not vary significantly across age categories, marital status, education levels, work status, and residence place across Delhi and NCR (*p* ≤ .05). A non-significant variation in attitudes regarding the protocols and control behaviors across the socio-demographic categories reflects an overall community’s Attitude toward the steps undertaken for virus control.

### Assessment of Practices

In this study, practices toward COVID-19 were measured through five questions: avoidance of social events, markets, crowded places, avoidance of handshaking, and practicing proper hand hygiene. Every sound practice was marked as “1,” and every bad practice was allotted “0.” The practice scores ranged from 0 to 5. The mean score of practice was 3.99 (*SD* = 1.03, maximum score = 5, minimum score = 1; Table 4).

**Table 4.** Responses to Practices Responses About COVID-19.

Statements	Yes (%)	No (%)
Have you recently attended a large-scale social event?	252 (25.14)	750 (74.85)
Have you lately visited a crowded location, such as a market?	480 (47.90)	522 (52.09)
Have you recently abstained from cultural practices such as handshakes?	879 (87.72)	123 (12.27)
Have you been utilizing social distancing techniques?	954 (95.20)	48 (4.79)
Have you recently frequently cleansed your hands with soap and water for at least 40 seconds, particularly after entering a public space or after blowing your nose, coughing, or sneezing?	901 (89.92)	101 (10.07)

In response to the first question about skipping social gatherings, 74.85% of respondents said they had done so. In contrast, an overwhelmingly large proportion of respondents admitted to engaging in social isolation. When asked about recent visits to crowded markets, approximately 48% of respondents said they had been to markets. In addition, approximately 12.27% of respondents admitted they had not avoided handshakes. Practices differed significantly by gender classification (*p* ≤ .05). The average score for practice was 3.90 for male respondents and 4.09 for female respondents. The scores for practice varied significantly across education, employment, and residence categories (*p* ≤ .05). Respondents with a postgraduate education demonstrated the highest average practice score (4.22), followed by those with a high school education or less. The respondents with the highest practice scores (4.22) were retirees, followed by government workers (4.32). There were significant associations between practicing good hand hygiene and gender, place of residence, and profession. Females, residents of southwest Delhi, and the elderly were more likely to practice proper hand hygiene. The practice scores did not vary significantly by marital status or age group.

### Relationship Between Respondent’s Knowledge, Attitudes, and Practices

Knowledge is a prerequisite for adopting a protective mentality and behavior toward a prevalent illness. Studies from around the world indicate that mere knowledge of a disease does not influence the population’s attitudes or behaviors (Saqlain et al., 2020; Shi et al., 2020; Suvvari et al., 2021; Wadhwani et al., 2020). In addition, despite adequate knowledge, the attitude was not always positive, necessitating additional education to explain the importance of developing a positive attitude and engaging in ongoing preventative practices aimed at reducing the incidence of HIV infection and transmission (Nwagbara et al., 2021). In this study, Pearson’s correlation was used to determine whether there is a significant correlation between knowledge, attitudes, and practices (Table 5).

**Table 5.** Correlation Between Knowledge, Attitude, and Practices Scores.

	Knowledge score	Attitude score
Knowledge score		
Pearson correlation	1	.068*
Sig. (two-tailed)		0.032
Attitude score		
Pearson correlation	.068*	
Sig. (two-tailed)	0.032	
	Knowledge score	Practice score
Knowledge score		
Pearson correlation	1	.042
Sig. (two-tailed)		0.187
Practice score		
Pearson correlation	.042	
Sig. (two-tailed)	0.187	
	Practice score	Attitude score
Practice score		
Pearson correlation	1	.166**
Sig. (two-tailed)		<0.001
Attitude score		
Pearson correlation	.16**	
Sig.(two-tailed)	<0.001	

* Csorrelation is significant at the .05 level (two-tailed)

** Correlation is significant at the .01 level (two-tailed)

Similarly, in this study, a significant positive Pearson’s correlation was found between knowledge and attitude (*p* ≤ .05) and between attitude and practice (*p* ≤ .01). There was no correlation between knowledge and practice scores. This is because the adoption of preventive practices is determined by underlying socio-economic realities, such as population density, past government experiences controlling diseases, etc. (Bates et al., 2021; Nwagbara et al., 2021; Table 6).

**Table 6.** Comparison of Knowledge, Attitude, and Practice Scores Among Different Demographic Variables.

Variables	Knowledge scores			Attitude scores			Practice scores		
	*M* ± *SD*	*t/F* Value	*p* Value	*M* ± *SD*	*t/F* Value	*p* Value	*M* ± *SD*	*t/F* Value	*p* Value
Gender									
Male	13.14 ± 1.56	0.20	.69	27.11 ± 2.72	0.13	.08	3.90 ± 1.05	0.00	.00
Female	13.14 ± 1.56			27.37 ± 2.67			4.09 ± 0.99		
Age-group (years)									
18–29	13.05 ± 1.61	2.48	.04	27.22 ± 2.82	2.70	.02	3.94 ± 1.04	0.69	.059
30–39	13.16 ± 1.63			26.71 ± 2.91			4.03 ± 0.94		
40–49	13.43 ± 1.42			27.46 ± 2.35			4.05 ± 1.04		
50–59	12.96 ± 1.62			27.65 ± 2.25			3.97 ± 1.13		
60 and above	13.13 ± 1.39			27.24 ± 2.87			4.08 ± 0.93		
Work status									
Government	13.26 ± 1.39	4.23	.00	27.42 ± 3.00	1.51	.19	4.32 ± 0.77	3.81	.00
Private sector	13.5 ± 1.50			26.79 ± 2.64			3.90 ± 1.00		
Retiree	14.5 ± 0.52			28.16 ± 1.60			4.5 ± 0.83		
Self-employed	13.24 ± 1.43			27.53 ± 2.45			4.01 ± 1.01		
Unemployed	13.02 ± 1.61			27.26 ± 2.68			3.95 ± 1.06		
Marital status									
Married	13.00 ± 1.61	10.93	.00	27.21 ± 2.78	0.17	.83	3.96 ± 1.04	2.49	.08
Unmarried	13.46 ± 1.31			27.28 ± 2.53			4.10 ± 0.95		
Others	13.85 ± 2.19			27.52 ± 2.01			3.76 ± 1.22		
Education level									
High school or below	13.14 ± 1.76	3.31	.19	27.23 ± 2.76	0.93	.42	4.10 ± 1.02	4.37	.00
Graduate	13 ± 1.47			27.12 ± 2.71			3.89 ± 1.09		
Postgraduate	13.41 ± 1.51			27.36 ± 2.66			4.22 ± 0.89		
Others	13.30 ± 1.17			27.64 ± 2.39			3.98 ± 0.90		

In the current study, attitudes did not differ significantly across age groups, marital status, education levels, employment status, and study area residential localities (*p* ≤ .05). Again, this was a significant finding that was consistent with findings from other studies in populations from various countries (Ferdous et al., 2020; Saqlain et al., 2020; Srichan et al., 2020). Knowledge and practice scores varied considerably by educational level and employment status. The average knowledge scores ranged from a minimum of 5 to a maximum of 17 points. Similarly, knowledge scores varied considerably with age. The respondents aged 50 years and older had the lowest average knowledge score. This may be a result of the digital divide in the country, where the elderly has limited access to online health information resources and are therefore unaware of the most recent COVID-19 information. Correct COVID-19 knowledge rates varied considerably, indicating that while some participants had extensive knowledge of the condition, others lacked such knowledge. Practice scores varied significantly by gender, education, employment, and residential status categories (*p* ≤ .05). Adopting preventive measures merits further explanation. Regarding certain measures, such as hand washing for a specified period of time, the results were strikingly similar to those of other studies conducted in other parts of the world (Ahdab, 2020; Zegarra-Valdivia et al., 2020; Zhong et al., 2020). A 54.8% of participants in the study conducted in northern Thailand did not routinely use soap when washing their hands (Srichan et al., 2020). In the present study, significant associations were found between good practice scores and sample gender.

## Discussion

In the present study, knowledge, attitude, and practice regarding COVID-19 in Delhi and the National Capital Region (NCR) were evaluated. As stated previously, knowledge is without a doubt an essential component of self-care promotion. Nonetheless, knowledge alone cannot guarantee protective behavior or result in a positive attitude. The absence of a significant correlation between respondents’ knowledge and practice scores indicates the same. Multiple factors influence a person’s knowledge of disease and the likelihood of adopting a positive outlook or engaging in healthy behavior. Critical determinants include the severity of the disease; vulnerability; exposure to it; the benefits of adopting a positive attitude and behavior, and obstacles that may prevent an individual from adopting positive changes. It makes sense that there is a significant correlation between attitude and practice scores. Respondents with favorable attitudes toward the protocols and stringent measures adopted by governments in regard to COVID-19 would also engage in these actions. They would also take measures to help prevent the virus’s spread. They would also take measures to help prevent the virus’s spread.

The findings reveal a sizeable number of socio-demographic factors that influence KAP and should prove beneficial while planning health education programs about other emerging infectious diseases. We found that, during the study period, 77.29% of the participants had average knowledge of COVID-19. This finding is consistent with prior research indicating that the Indian people has a sufficient level of knowledge of outbreaks such as MERS and Influenza (Kant, 2015; Koul et al., 2017). The study revealed that a vast majority of subjects described some of the most common symptoms associated with COVID-19 There was only a very small minority that was unaware of any of the symptoms, similar to other studies elsewhere (Ferdous et al., 2020; Zegarra-Valdivia et al., 2020). In the current study, about 77.29% of the respondents had average knowledge of the virus which was lower than what was found in a study conducted among the Chinese population, who had an average knowledge score of 90% (Zhong et al., 2020). This indicates a significant education gap, likely reflecting suboptimal public health information and dissemination regarding COVID-19. Thus, it is necessary to customize health officials’ and other media outlets’ information about the condition to address the diverse character of the factors contributing to decreased knowledge (Ferdous et al., 2020). However, there was no significant difference in mean knowledge scores for different gender categories. This was inconsistent with the findings of other studies conducted elsewhere where gender was a significant factor impacting the knowledge of the virus (Srichan et al., 2020; Zhong et al., 2020). Also, about 91% of the participants had a positive attitude toward COVID-19. Further, attitude scores across gender roles were not significantly different; gender played a considerable role in mean practice scores. This result is consistent with the findings of studies conducted in China, which also found that gender had an effect on practice scores (Shi et al., 2020; Zhong et al., 2020). In this study, 84% of respondents strongly agreed that transmission of COVID-19 could be prevented by implementing universal precautions as recommended by the WHO and Ministry of Health. Similar conclusions were reached by other studies conducted in various nations regarding adherence to the universally accepted precautions outlined by health agencies (Saqlain et al., 2020; Zhong et al., 2020).

Across the globe, women were much more likely than men to Globally, women are significantly more likely than men to engage in preventive behavior. This is an important finding that may have a substantial effect on the success of home dissemination of preventative guidelines, as targeting women may ultimately result in improved household behavior. The majority of participants in the current study reported taking preventative measures, such as avoiding crowded areas and practicing proper hand hygiene. This demonstrates that individuals have a general tendency to modify their behavior in response to the COVID-19 epidemic. A large percentage of respondents, however, reported visiting crowded markets. Out of the four established COVID-19 protective behaviors—mask-wearing, self-isolation, physical distancing, and handwashing—only not wearing a mask is punishable. Almost every nation has declared fines of varying amounts since the virus’s spread last year. During epidemic outbreaks, the states increased the fine amount. Between high COVID restriction time (HCRT) and relaxed COVID restriction time (RCRT), the fine amount decreased. Additional exemptions were granted, such as the mask not being required in private vehicles, etc. Prior to the study period, numerous locations in the study area reported large crowds during the festival season. Following this, there was an increase in the number of COVID-19 cases reported in New Delhi. According to a wide-ranging news report from the region, large crowds gathered at various marketplaces during the festival season in multiple cities throughout South Asia. People did not practice social distance (Economic Times, n.d.). Such behavior was observed all over the world and was attributed to the human propensity to avoid risk-taking (Parrish, 2020). Introducing new precautions into everyday life can be taxing. Then, some desire to take risks without fear of the repercussions. In a variety of studies, psychologists have recommended practices to prevent COVID-19 “safety fatigue” or “burnout” (Parrish, 2020; Zhan et al., 2020). Such conduct may prove fatal in our fight against COVID-19. The Indian government has periodically warned its citizens against such complacency.

The outbreak of COVID-19 has exacerbated anxiety and stress in people subjected to the real or perceived threat of the virus. Various studies have been conducted to assess the knowledge, attitude, and practices (KAP) of residents of different countries and regions of the world (Al-Hanawi et al., 2020; Azlan et al., 2020; Ferdous et al., 2020; Hussain et al., 2020; Joseph et al., 2021; Reuben et al., 2020; Shi et al., 2020; Zhong et al., 2020). COVID-19 exposed the health care workers (HCWs) around the world to address medical exigencies of mammoth proportions which were hereto unimaginable. Studies were conducted to assess the psychological responses of HCWs during the epidemic, determine the stressors and identify possible ways to cope with the unwarranted situation (Peng et al., 2020; Slama et al., 2021). Such studies have also been conducted in India in different regions and also among the population of different professions, for example, health care workers, medical students, etc (Agarwal et al., 2020; Dkhar et al., 2020; Gopalakrishnan et al., 2021; Maheshwari et al., 2020; Padmanaban et al., 2021; Wadhwani et al., 2020). A similar cross-sectional study for a sample of 1,292 respondents drawn from the Indian population revealed 81% of the respondents had average knowledge of the virus, which was slightly higher than the findings of this study Upon comparing the attitudes scores of various studies, it was discovered that 77% of the respondents in the Indian population have average attitudes and 83.5% of the respondents showed good practices scores, whereas in this study, approximately 91% of respondents had average attitudes and approximately 95.2% of respondents admitted to having followed the practice of social distancing.

The study revealed that even after almost a year into this, pandemic respondents had mixed responses about the need for testing persons without any history of contact. Such confusion was also revealed about the requirement for hospitalization in each case. It was understandable that studies conducted during the early phase of the lockdown also showed such a lack of knowledge (Wadhwani et al., 2020). At that time, even the medical personnel and hospitals were unclear about their roles. But, a year later, such confusion persists, and this is a lacuna to be bridged with better awareness and communication programs. There is also some confusion about the infection source. About 23% of respondents accepted or were unaware that eating or being in contact with animals does not propagate or result in the virus spread. The highest mixed response was seen regarding antibiotics as an effective treatment for COVID-19.

India is a diverse country with vastly different income levels, education levels, and other socio-economic parameters. As a result, it is projected that the population’s levels of knowledge, attitude, and prevention will likewise vary significantly. It is very likely that sections of the population with little or no access to the internet or who live in far-flung regions may display reduced KAP. Thus, for the dissemination of information regarding the virus and to increase the acceptability of the available vaccine, suitable initiatives would need to be implemented.

## Limitations

This study had some limitations. This study observed a cross-sectional study design thereby establishing causal inferences may not be possible. As compared to face-to-face interviews, self-reporting has limitations and can be inflicted with multiple biases. The sampling technique used was snowball sampling, and the questionnaires were disseminated through social media platforms through the personal and professional networks of researchers. As a result, bias is possible, as disadvantaged populations may have been excluded from the study. Since the study participants were the literate community that understood English and were active on social media, the study findings can only be generalized possibly to urban Delhi (or other metropolitan cities of the country) population. Since a limited number of questions were included in the study to measure the level of knowledge, attitude, and practice, there is a pertinent need to conduct multiple assessments including all aspects of KAP toward COVID-19. KAP as a study instrument can always be made more robust by including yet better questions regarding aspects that affect the Knowledge, Attitude, and practices of the population about COVID-19 to determine the actual extent of KAP in the general population. Additionally, this study excluded information about the population’s knowledge, attitude, and behavior regarding the COVID-19 vaccine, which represents a major opportunity for minimizing COVID-19 transmission and is proving to be a critical step in addressing the global COVID-19 pandemic (Alexander et al., 2021).

## Conclusions

This study was conducted almost one year after the first case of COVID-19 was detected in the world. It provided a comprehensive examination of Delhi and NCR residents’ knowledge, Attitude, and practices toward the virus spread. Later time through the pandemic has shown that consistent spread of messages regarding the virus, its spread, control measures adopted, and precautions expected of the public has gone a long way in assuring the masses, controlling their anxiousness, and curbing the large spread of fake news about the virus. Similar effective dissemination of information and knowledge about the effectiveness of the vaccine has improved its adoption among the public in different countries of the world. Specific awareness programs, effective communication measures both about the virus and now importantly about the vaccine, its availability, and efficacy is going a long way in building the communities’ resilience. For future research, estimating the acceptability of various vaccines, the willingness of the population to pay for such vaccines will go a long way in modulating effective vaccination strategies by various governments. Several researchers (Alexander et al., 2021; Das et al., 2021; Qin et al., 2021) have embarked their efforts on such critical issues which will have significant impacts on devising efficient investments in health protection measures in different parts of the world.
